# A prospective randomized trial of content expertise versus process expertise in small group teaching

**DOI:** 10.1186/1472-6920-10-70

**Published:** 2010-10-14

**Authors:** Adam D Peets, Lara Cooke, Bruce Wright, Sylvain Coderre, Kevin McLaughlin

**Affiliations:** 1 Division of Critical Care Medicine, Centre for Health Education Scholarship and Centre for Health Evaluation and Outcome Sciences, University of British Columbia, Vancouver, Canada; 2 Department of Clinical Neurosciences and Office of Undergraduate Medical Education, Calgary, Canada; 3 Department of Family Medicine and Office of Undergraduate Medical Education, Calgary, Canada; 4 Division of Gastroenterology and Office of Undergraduate Medical Education, Calgary, Canada; 5 Division of Nephrology and Office of Undergraduate Medical Education, Calgary, Canada; 6 ADP was a member of the Office of Undergraduate Medical Education at the University of Calgary at the time of this study

## Abstract

**Background:**

Effective teaching requires an understanding of both *what *(content knowledge) and *how *(process knowledge) to teach. While previous studies involving medical students have compared preceptors with greater or lesser content knowledge, it is unclear whether process expertise can compensate for deficient content expertise. Therefore, the objective of our study was to compare the effect of preceptors with process expertise to those with content expertise on medical students' learning outcomes in a structured small group environment.

**Methods:**

One hundred and fifty-one first year medical students were randomized to 11 groups for the small group component of the Cardiovascular-Respiratory course at the University of Calgary. Each group was then block randomized to one of three streams for the entire course: tutoring exclusively by physicians with content expertise (n = 5), tutoring exclusively by physicians with process expertise (n = 3), and tutoring by content experts for 11 sessions and process experts for 10 sessions (n = 3). After each of the 21 small group sessions, students evaluated their preceptors' teaching with a standardized instrument. Students' knowledge acquisition was assessed by an end-of-course multiple choice (EOC-MCQ) examination.

**Results:**

Students rated the process experts significantly higher on each of the instrument's 15 items, including the overall rating. Students' mean score (±SD) on the EOC-MCQ exam was 76.1% (8.1) for groups taught by content experts, 78.2% (7.8) for the combination group and 79.5% (9.2) for process expert groups (p = 0.11). By linear regression student performance was higher if they had been taught by process experts (regression coefficient 2.7 [0.1, 5.4], p < .05), but not content experts (p = .09).

**Conclusions:**

When preceptors are physicians, content expertise is not a prerequisite to teach first year medical students within a structured small group environment; preceptors with process expertise result in at least equivalent, if not superior, student outcomes in this setting.

## Background

Teaching is a knowledge-based skill: we can't teach what we don't know. But content expertise alone does not guarantee effective teaching. Rather, this requires an understanding of both *what *(content knowledge) and *how *(process knowledge) to teach[1]. So, can process knowledge compensate for deficient content knowledge?

Several studies involving problem-based learning (PBL) tutorials have highlighted teaching differences between preceptors who either have or do not have content expertise. Students in small groups tutored by content experts tend to generate more learning issues and participate in additional self-directed studying [2,3]. However, they also report that content experts are more likely to *direct *learning, whereas non-experts tend to *facilitate *learning [4-7]. But does this difference in teaching styles affect learning outcomes?

Davis *et al. *[8] found PBL preceptors with content expertise were not only rated as better teachers than their non-expert peers, but their students had better learning outcomes. Schmidt *et al. *[1,3] subsequently confirmed this. However, in 1994 Davis *et al. *[9] published another study that appeared to contradict their earlier work and suggested that the benefit of content expertise disappears when highly structured cases are used. Other groups confirmed this new finding and it was proposed that highly structured cases may reduce the knowledge gap between expert and non-expert preceptor and/or reduce the dependency of students' learning on their preceptors' knowledge [10,11]	.

Most studies in this area have compared preceptors with greater or lesser content knowledge. But what about the *how *of teaching; does better process knowledge improve learning outcomes? Ideally preceptors should have both content and process expertise, but this constellation is not commonly seen. Indeed, the interest in preceptors without content expertise arose from a decision to move towards more resource intensive curricula, such as problem-based learning, where the demand for preceptors outstripped the supply of content experts. Perhaps it is now more relevant to ask if process expertise can compensate for deficient content expertise.

Therefore, the objective of our study was to evaluate the effect of tutors' content or process expertise on learning outcomes. Our hypothesis was that process expertise would compensate for a relative lack of content expertise and lead to equivalent learning outcomes for students.

## Methods

At the University of Calgary medical school we have a three year clinical presentation curriculum [12]. During the first two years, students undertake seven sequential systems-based courses that utilize a combination of lectures and small group case-based sessions to present the majority of the curricular content. These learning activities are supplemented with laboratory-based sessions that explore relevant anatomy and pathology and also bedside teaching sessions that enable students to see firsthand the conditions they are learning about in real patients. At the beginning of the first year we randomly allocate students to one of 11 groups, each with 13-14 students, for small group teaching sessions.

We conducted this study using the 12 week long Cardiovascular-Respiratory course, which is the third course of the first year curriculum. The course includes 21 small group sessions, each lasting two hours; student attendance for these is mandatory. Prior to each session students and preceptors received the cases and questions to be covered. During the session, the students progressed through each case and discussed the questions that had been distributed previously and any new questions that may have arisen. The preceptor served as a resource to help guide the discussion and provide a contextual basis for the material being covered. In the preceptor's guide we also included suggested answers from a content expert. During the study we gave identical material to all preceptors.

This was a prospective randomized trial that was approved by the Conjoint Health Research Board at the University of Calgary. Our intervention was two different types of preceptors for small group sessions: content experts or process experts. Each of our content experts either had, or was training for, Royal College of Physicians and Surgeons of Canada (RCPSC) subspecialty certification in the clinical domains of study (Cardiology or Respirology), and their clinical practice was entirely in these domains. None of our process experts had, or were training for, subspecialty certification in the clinical domains of study, but were part of the Master Teacher program at the University of Calgary. This Program provides a yearly salary in exchange for 340 hours of teaching per year. Physicians identified by their peers and students as superior educators go through an interview process and if accepted into the Program, complete the Teaching Scholars in Medicine Certificate Program (TSIMP), an eighty hour University of Calgary accredited certificate program designed to foster expert instructional skills. Master Teachers subsequently teach in every Course during the first 2 years of our curriculum. Each of our 11 Master Teachers participated in the study. All are 'generalists' by training (six Internists, four Family Physicians, and one Emergency Medicine Physician). While all of the content experts had previously taught small group sessions, none had been part of the Master Teacher Program nor had any taken the TSIMP or similar programs.

We randomly allocated each of the 11 small groups to one of three streams for the entire length of the course: 1) tutored exclusively by physicians who were content experts (n = 5 groups); 2) tutored exclusively by physicians who were process experts (n = 3 groups); and 3) tutored by either a content expert (11 sessions) or a process expert (10 sessions) (n = 3 groups). The inequality in the number of groups in each of the streams was due to the limited number of process experts we had available.

We included the group exposed to both types of experts because it represented an intermediate position between content and process expertise, thus allowing us to look for a gradient in learning outcomes [13], and because it represented the status quo for the students after the study period. While small group sessions in the systems-based courses had traditionally been facilitated exclusively by Specialists with content expertise in the topic being discussed, when the class size more than doubled over a 4 year period, there was no longer an adequate number of Specialists to facilitate the groups. Therefore, the Master Teacher Program was created to fill the vacancies and as a result, over a typical course, students would have groups facilitated by both content experts and process experts.

To assess the process expertise of all preceptors, we had students rate their teaching skills at the end of each small group session using (with permission) a modified version of the tool developed and validated by the Stanford University Faculty Development Program [14]. All preceptors received a copy of the tool prior to the start of the course.

We used student performance on the certifying end-of-course multiple choice question (EOC-MCQ) examination as a measure of learning outcomes. This evaluation is based upon an evaluation blueprint that is congruent with the learning objectives and learning experiences of the course [15].

We used Cronbach's alpha to evaluate the internal reliability of the tutor evaluation tool and the EOC-MCQ. To assess our randomization process we performed one-way analysis of variance (ANOVA) comparing the mean scores for each group on the EOC-MCQ examinations for the two courses that preceded the Cardiovascular-Respiratory course (Gastrointestinal/Blood course and the Musculoskeletal course).

We also used one-way ANOVA to compare the performance on the EOC-MCQ examination for students in each of the three groups during the Cardiovascular-Respiratory course. We then employed multiple linear regression to evaluate the impact of content and process expertise on learning outcomes. The dependent variable was score on the EOC-MCQ, and independent variables were the group to which each student was assigned and mean score for each student on the EOC-MCQ examination for courses 1 and 2. We used both SPSS version 16.0 and STATA 8.0 for our statistical analyses.

## Results

One hundred and fifty one students completed the Cardiovascular-Respiratory course, of which 68 were randomized to content expert groups, 41 to process expert groups, and 42 to the groups taught by both content and process experts. Throughout the course, the 11 Master Teachers and 60 content experts were randomly assigned to teach sessions within their small group type. No group had the same preceptor more than 3 times over the entire course.

Students completed 2359 small group preceptor evaluation forms. The internal reliability of this evaluation form was 0.95. Students rated Master Teachers significantly higher on each of the instrument's 15 items, including the overall rating (Table 1).

**Table 1:** Teaching evaluations for process and content experts

Item	Process Experts	Content Experts	p value
Listened to learners	4.83 [4.80-4.85]*	4.68 [4.65-4.71]	<.001

Encouraged learners to participate actively in discussion	4.76 [4.73-4.79]	4.62 [4.59-4.66]	<.001

Expressed respect for learners	4.86 [4.84-4.88]	4.70 [4.67-4.73]	<.001

Encouraged learners to bring up problems	4.79 [4.76-4.82]	4.64 [4.61-4.68]	<.001

Called attention to time	4.61 [4.56-4.66]	4.45 [4.40-4.49]	<.001

Avoided digressions	4.58 [4.53-4.62]	4.47 [4.42-4.51]	<.01

Effectively dealt with disruptive students	4.74 [4.69-4.80]	4.61 [4.54-4.68]	<.01

Stated goals/objectives of the session clearly and concisely	4.63 [4.58-4.67]	4.42 [4.38-4.46]	<.001

Stated relevance of goals/objectives to learners	4.56 [4.51-4.60]	4.42 [4.38-4.46]	<.001

Repeated goals and objectives periodically	4.43 [4.38-4.49]	4.26 [4.21-4.31]	<.001

Used whiteboard or other visual aids	4.74 [4.70-4.78]	4.55 [4.51-4.59]	<.001

Referred to relevant schemes for clinical presentations	4.68 [4.64-4.72]	4.53 [4.49-4.57]	<.001

Provided ample opportunity for students to ask questions	4.83 [4.81-4.86]	4.70 [4.67-4.73]	<.001

Helped students draw connections between the clinical presentation and relevant science/physiology/anatomy	4.76 [4.72-4.79]	4.65 [4.62-4.68]	<.001

Overall, this preceptor was an effective small group facilitator	4.78 [4.74-4.81]	4.62 [4.58-4.65]	<.001

*Data presented as mean score on 5 point scale, followed by 95% confidence interval

There was no difference in prior academic performance for students in each of the small group types. The mean scores (±SD) for the EOC-MCQ examinations for the first two courses of the first year curriculum were 76.6% (6.9), 78.1% (6.9) and 77.6% (7.1), for the content expert, content and process expert and the process expert groups, respectively (p = .71).

For the Cardiovascular-Respiratory EOC-MCQ examination, Cronbach's alpha was 0.78 and mean score for the entire class was 77.6% (8.4). The mean scores were 76.1% (8.1), 78.2% (7.8) and 79.5% (9.2) for the content expert, content and process expert and the process expert groups, respectively (p = .11).

By linear regression we found no effect of content expertise on performance (regression coefficient -2.6 [-5.6, 0.4], p = .09). Process expertise was, however, associated with a small, but statistically significant, improvement in performance (regression coefficient 2.7 [0.1, 5.4], p < .05).The difference in performance associated with process expertise had a small to medium effect size (Cohen's *d *= .40).

## Discussion

In this study we compared the effect of different types of preceptor expertise - content versus process - on student learning in a small group setting. We found no significant differences in learning outcomes between groups where preceptors were exclusively content experts, process experts, or a mixture of both. However, when we studied the effects of content and process expertise separately we found that process expertise was associated with a small, but significant, improvement in learning outcomes, whereas content expertise was not. So why are our results at odds with earlier studies that found improved learning outcomes when preceptors were content experts [1,3,8]?

Our discrepant results may be due to smaller content knowledge gap between our two different types of preceptors. Whereas previous studies have used process experts from non-physician backgrounds such as social work and the basic sciences, ours were practicing physicians from generalist specialties. Therefore, they would likely encounter the content covered in the Cardiovascular-Respiratory course during their clinical practice, albeit with reduced frequency. We also provided all preceptors with the same teaching content rather than expect them to create their own. This interpretation is consistent with previous studies that also noted the effects of content expertise are reduced if non-expert preceptors are provided with structured teaching content [9-11].

But there is another explanation for our discrepant results: the effect of process expertise. Early studies focused on content expertise and categorized preceptors into those who had more or less of it. By contrast, our preceptors without content expertise had process expertise, and not only by reputation; they also demonstrated their greater process expertise during the course, as reflected in the results of the student evaluations. Our findings suggest that process expertise can compensate for, or even surpass, deficient content expertise. There are a number of underlying mechanisms that help explain why this may be the case.

The relationship between content expertise, teaching performance, and learning outcomes is non-linear. In fact, depending upon the group of learners, content expertise may hinder teaching performance. Schmidt defines 'cognitive congruence' as "the ability to express oneself in the language of the students", and suggests that teaching cannot be effective without this [1]. But content experts do not usually think in the language of students. Ericsson has shown that with increasing expertise cognitive processes become automated [16]. Similarly, Peyton describes specialists as functioning with 'unconscious competence', but having to return to the level of 'conscious competence' to teach students [17]. Consequently, achieving cognitive congruence, particularly with novice learners, may be more challenging for content experts.

Several groups have also reported that content experts tend to direct learning more than preceptors without content expertise [4-7]. Increased preceptor direction can lead to learners being more passive during the small group sessions. Eagle *et al. *[2] and Schmidt *et al. *[3] also noted that students identified more learning issues when their preceptor was a content expert. Given the capacity limitations of working memory, extra information introduced by content experts may result in a larger cognitive load and less effective learning [18]. This is particularly so when teaching learners in whom new content generates a larger intrinsic cognitive load [19]. It is conceivable that our process experts, because of their adequate but relatively limited relevant knowledge, restrict themselves to the core material. In this way process experts may facilitate a more succinct and cognitively less demanding session, resulting in greater long-term retention of key concepts.

Yet, our results should not be interpreted as implying that knowing *how *to teach in a small group setting is more important than knowing *what *to teach. Hay *et al. *[5] demonstrated that if a preceptor lacks the appropriate clinical knowledge then learning outcomes are poorer - even if this preceptor has higher ratings on teaching performance - suggesting that large gaps in content knowledge cannot be bridged by process expertise alone.

Our study has limitations. While the difference in examination scores between the two groups is statistically significant, it is numerically small. However, it is important to note that the intervention targeted only one aspect of how students learned course material and did not alter their educational experience in lectures, in the laboratory, at the bedside or, most importantly, how they studied the material on their own. Since medical students are typically already high achievers from an academic standpoint, even small changes in academic performance may be an important finding. This may be even more significant when we consider that the comparison group of content experts still provided the students with an excellent educational experience (overall rating of 4.6 out of 5.0 on the student evaluations). That being said, the relevance of a 2.7% difference in written test scores when it comes to future clinical practice remains unclear. Further studies assessing both short and long-term outcomes are required.

Additionally, our results may not generalize to other curricula because of the three year clinical presentation curriculum with highly structured small group sessions that is used at the University of Calgary. The course we chose also covered topics with which our process experts were quite familiar; results may have been different if less common clinical presentations were studied or process experts without generalist training were selected. In addition, the structured nature of the small group sessions, including the use of preceptor guides, may have reduced the importance of content expertise as a characteristic of a successful preceptor. Finally, our preceptors were either content or process experts, but not both. Do 'dual expert' preceptors improve learning further? We can't comment on this as we intentionally targeted a gap in process expertise, but this is clearly a question that should be addressed in future studies.

## Conclusions

Our results suggest that preceptors with process expertise result in at least equivalent, if not superior, student outcomes when compared to those with content expertise. Based on the characteristics of a small group learning session, including format, type of learner and type of content covered, there likely exists an ideal combination of content and process expertise that a preceptor should possess in order to optimize students' learning outcomes. Further research is needed in order to determine what the ideal combinations of expertise are and then determining how best to facilitate preceptors' acquisition of the appropriate level of knowledge and skills.

## Competing interests

The authors declare that they have no competing interests.

## Authors' contributions

ADP contributed to the conception and design of the study, acquisition of data, interpretation of data, drafting of manuscript and revising it critically for important intellectual content. LC contributed to the conception and design of the study, interpretation of data, drafting of manuscript and revising it critically for important intellectual content. BW contributed to the conception of the study, interpretation of data, drafting of manuscript and revising it critically for important intellectual content. SC contributed to the conception and design of the study, interpretation of data, drafting of manuscript and revising it critically for important intellectual content. KM contributed to the conception and design of the study, acquisition of data, interpretation of data, drafting of manuscript and revising it critically for important intellectual content. All authors read and approved the final manuscript.

## Pre-publication history

The pre-publication history for this paper can be accessed here:

http://www.biomedcentral.com/1472-6920/10/70/prepub
